# Impaired osteogenesis of T1DM bone marrow-derived stromal cells and periosteum-derived cells and their differential in-vitro responses to growth factor rescue

**DOI:** 10.1186/s13287-017-0521-6

**Published:** 2017-03-11

**Authors:** Tera M. Filion, Jordan D. Skelly, Henry Huang, Dale L. Greiner, David C. Ayers, Jie Song

**Affiliations:** 1 Department of Orthopedics & Physical Rehabilitation, University of Massachusetts Medical School, 55 Lake Avenue North, S4-827, Worcester, MA 01655 USA; 2 Department of Molecular Medicine, Diabetes Center of Excellence™, University of Massachusetts Medical School, 55 Lake Avenue North, Worcester, MA 01655 USA

**Keywords:** Type 1 diabetes, Bone marrow-derived stromal cell, Periosteum-derived cell, Osteogenesis, Hyperglycemia, Growth factor rescue, Bone morphogenetic protein-2/7 heterodimer, Insulin-like growth factor-1

## Abstract

**Background:**

Poor bone quality, increased fracture risks, and impaired bone healing are orthopedic comorbidities of type 1 diabetes (T1DM). Standard osteogenic growth factor treatments are inadequate in fully rescuing retarded healing of traumatic T1DM long bone injuries where both periosteal and bone marrow niches are disrupted. We test the hypotheses that osteogenesis of bone marrow-derived stromal cells (BMSCs) and periosteum-derived cells (PDCs), two critical skeletal progenitors in long bone healing, are both impaired in T1DM and that they respond differentially to osteogenic bone morphogenetic proteins (BMPs) and/or insulin-like growth factor-1 (IGF-1) rescue.

**Methods:**

BMSCs and PDCs were isolated from Biobreeding Diabetes Prone/Worcester rats acquiring T1DM and normal Wistar rats. Proliferation, osteogenesis, and adipogenesis of the diabetic progenitors were compared with normal controls. Responses of diabetic progenitors to osteogenesis rescue by rhBMP-2/7 heterodimer (45 or 300 ng/ml) and/or rhIGF-1 (15 or 100 ng/ml) in normal and high glucose cultures were examined by alizarin red staining and qPCR.

**Results:**

Diabetic BMSCs and PDCs proliferated slower and underwent poorer osteogenesis than nondiabetic controls, and these impairments were exacerbated in high glucose cultures. Osteogenesis of diabetic PDCs was rescued by rhBMP-2/7 or rhBMP-2/7 + rhIGF-1 in both normal and high glucose cultures in a dose-dependent manner. Diabetic BMSCs, however, only responded to 300 ng/nl rhBMP-2/7 with/without 100 ng/ml rhIGF-1 in normal but not high glucose osteogenic culture. IGF-1 alone was insufficient in rescuing the osteogenesis of either diabetic progenitor. Supplementing rhBMP-2/7 in high glucose osteogenic culture significantly enhanced gene expressions of type 1 collagen (Col 1), osteocalcin (OCN), and glucose transporter 1 (GLUT1) while suppressing that of adipogenic marker peroxisome proliferator-activated receptor gamma (PPARγ) in diabetic PDCs. The same treatment in high glucose culture only resulted in a moderate increase in Col 1, but no significant changes in OCN or GLUT1 expressions in diabetic BMSCs.

**Conclusions:**

This study demonstrates more effective osteogenesis rescue of diabetic PDCs than BMSCs by rhBMP-2/7 with/without rhIGF-1 in a hyperglycemia environment, underscoring the necessity to tailor biochemical therapeutics to specific skeletal progenitor niches. Our data also suggest potential benefits of combining growth factor treatment with blood glucose management to optimize orthopedic therapeutic outcomes for T1DM patients.

## Background

Type 1 diabetes (T1DM) is an inflammatory autoimmune disease characterized by the destruction of pancreatic beta cells resulting in insulin deficiency, hyperglycemia, and subsequent systemic health-related problems for those affected [1]. Approximately 1.25 million Americans are living with T1DM, which contributes to the $176 billion healthcare costs associated with diabetes in the USA in 2012 [2]. T1DM patients are at increased risks for bone fracture throughout life [3] due to compromised biomechanical integrity (increased brittleness) of bone [4], likely due to advanced glycation end product-mediated collagen crosslinking and inhibition of osteoblastic function [5, 6], and higher tendency for developing osteoporosis [7]. Meanwhile, bone healing in patients with poorly controlled T1DM is significantly compromised [8, 9], and growth hormone dysfunction, growth factor deficiencies, poor vascularization, neuropathy, and systemic and local inflammation have all been identified as potential contributors [8–10]. Increased adipogenicity in bone marrow [11], impaired osteoblastic activity/osteogenesis of bone marrow cells [12] and endochondral ossification [13], and high blood glucose-induced cellular senescence and apoptosis [14] have also been implicated in the bone pathology in T1DM.

Stem cells residing within different bone niche environments play distinct roles in bone healing, with bone marrow-derived stromal cells (BMSCs) contributing to intramembranous ossification and periosteum-derived cells (PDCs) contributing to endochondral ossification [15–17]. In traumatic long bone injuries, both the marrow cavity and periosteum are disrupted. The extent of injury to the bone and surrounding tissues could significantly compromise their effective healing [18]. Such a challenge is even more pronounced in the presence of DM. How skeletal progenitor BMSCs and PDCs are differentially impaired in T1DM, however, has not been carefully examined. This limitation has prevented effective therapeutic strategies for expediting T1DM bone healing from being developed, particularly the exceptionally challenging regenerative repair of critical size long bone injuries in diabetic patients.

T1DM rodents with impaired bone healing are valuable models for elucidating underlying cellular and molecular mechanisms of the orthopedic comorbidity of T1DM and developing potential bone healing therapies [19]. The widely used streptozotocin-induced T1DM rodents show decreased proliferation and osteogenic differentiation potential of BMSCs [12, 20, 21]. The systemic toxicity of streptozotocin [19], however, makes it difficult to carry out long-term evaluation of retarded bone healing in the chemically treated rodents because they tend to become very sick. Biobreeding Diabetes Prone/Worcester (BBDP) rats that spontaneously acquire the T1DM phenotype [22] more closely mimic clinically observed T1DM in that they spontaneously develop pancreatic insulitis similar to humans with T1DM, followed by selective beta cell destruction and diabetes [23]. They have been shown to exhibit compromised bone healing [24] and decreased osteoblastic activity [21], and thus are attractive alternatives. Systemic [25] and local [26] insulin deliveries were shown to rescue fracture healing in diabetic BBDP rats, but the restored blood glucose did not reflect the clinical scenarios faced by T1DM patients with poorly controlled glycemia. High doses of osteogenic growth factor bone morphogenetic protein-2 (3.6 μg rhBMP-2) were shown to promote the repair of noncritical size (3 mm) femoral defects in diabetic BBDP rats with poor glycemic control [27], but such a BMP-2 dose, when scaled up to the human, could present significant safety issues. Consistent with literature, we found that an osteoconductive synthetic bone graft in combination with 400 ng osteogenic rhBMP-2/7 heterodimer that functionally heals (with restoration of torsional strength) 5-mm critical femoral segmental defects in normal rats [28] was unable to bridge the same defects in diabetic BBDP rats with poorly controlled glycemia. Overall, safe and effective therapeutic strategies promoting the functional healing of critical long bone defects in T1DM BBDP rats with poorly controlled glycemia are still lacking, and the cellular mechanism underscoring such a challenge is yet to be fully elucidated.

Here we test the hypothesis that in-vitro osteogenesis of BMSCs and PDCs isolated from T1DM BBDP rats is impaired and that diabetic BMSCs and PDCs differentially respond to the rescue by potent osteogenic factor rhBMP-2/7 heterodimer and/or insulin growth factor rhIGF-1 in normal and high glucose cultures. Previous studies reported lower serum IGF-1 along with higher inflammatory cytokines and lower bone mineral density in T1DM patients with poor glycemic control than those with good glycemic control [29–31]. We first confirmed that the proliferation and osteogenesis of these diabetic BMSCs and PDCs were differentially impaired and these impairments were exacerbated in high glucose cultures. We then demonstrated that the osteogenesis of diabetic PDCs could be fully rescued by the supplementation of osteogenic growth factor rhBMP-2/7 heterodimer in both normal glucose and high glucose cultures in a dose-dependent manner. rhIGF-1 alone, however, could not sufficiently rescue the osteogenesis of diabetic PDCs in either normal or high glucose culture. The dual treatment of rhBMP-2/7 and rhIGF-1 resulted in most robust osteogenesis rescue of diabetic PDCs, although no apparent synergistic benefit beyond the sum of enhancements of the respective single factor treatments was observed. By contrast, we showed that the highly impaired osteogenesis of diabetic BMSCs could only be rescued by rhBMP-2/7 or rhBMP-2/7 + rhIGF-1 in normal glucose culture, but not in high glucose cultures. qPCR analyses corroborated drastically different responses by diabetic BMSCs and PDCs to rhBMP-2/7 treatment in cultures simulating the hyperglycemia microenvironment, suggesting the benefit of simultaneous glycemic control and growth factor therapy. This study underscores the need for tailoring therapeutic strategies to specific skeletal progenitor niches for improved therapeutic outcomes.

## Methods

### Maintenance of T1DM BBDP rats

All animal procedures were carried out per protocol A-1783 approved by the University of Massachusetts Medical School Institutional Animal Care and Use Committee. Four 4-week-old male BBDP rats (Biomedical Research Models, Inc., Worcester, MA, USA) were monitored for blood glucose (4-h fasting prior to measurement) and weight changes twice a week for the onset of T1DM. After two consecutive blood glucose readings of >250 mg/dl (onset as early as 9 weeks), the rats were considered to have acquired the T1DM phenotype. One rat was sacrificed upon confirmation of DM for isolation of freshly diabetic BMSCs and PDCs to investigate cell proliferation in high vs normal glucose cultures, while the others were given insulin implants (LinShin Canada, Inc.), delivered by a trocar to the back neck, to prevent excessive weight loss while maintaining blood glucose between 250 and 500 mg/dl emulating T1DM with poor glycemic control. The rats with more extended DM were maintained until ~100 days old before they were sacrificed for BMSC and PDC isolation for differentiation experiments.

### Isolation of diabetic and normal BMSCs and PDCs

BMSCs and PDCs were isolated as described previously [32] from long bones of either freshly diabetic BBDP rats, those with more extended T1DM (blood glucose maintained at 250–500 mg/dl, ~100 days), or healthy nondiabetic male Wistar rats (~100 days old; Charles River Lab). Wistar rats were chosen as normal nondiabetic controls because the BBDP rats were derived from the Wistar strain [23]. Although a small percentage of BBDP rats fail to develop the TIDM phenotype by 90 days, these rats are not appropriate as normal controls because they may simply have a later DM onset. Meanwhile, Biobreeding Diabetes Resistant/Worcester (BBDR) rats that are resistant to DM development have also been shown to exhibit abnormal cellular phenotypes [33], and thus are also unsuited as normal controls.

Briefly, marrow was flushed from femurs and tibias and pelleted before the red blood cells were lysed with sterile water. After centrifugation, the cell pellet was resuspended in expansion media (α-MEM without ascorbic acid with 20% FBS, 1% penicillin–streptomycin, and 1% l-glutamine), passed through a sterile swinney filter, and seeded (10 million/100-mm plate) for adherent culture. Media were changed on day 4 and three times a week thereafter. PDCs were isolated from femoral and tibial periosteum by enzymatic digestion. Briefly, the muscle was dissected to expose periosteal tissue, which was carefully scraped from the bone surface and digested in 2.5-mg/ml collagenase, type 2 (Worthington, Lakewood, NJ, USA) in α-MEM without ascorbic acid for 2 h under agitation at 37 °C. Cells were pelleted, resuspended in expansion media, and plated on 100-mm plates (two plates per leg). Medium was changed on day 4 and three times a week thereafter.

Given the general difficulty in expanding diabetic cells into higher passages, we used cells from no later than passage 2 for all experiments. To ensure consistency in the growth factor rescue experiments across all treatment conditions (with/without growth factors in six single/combination doses, normal and high glucose cultures; see Figs. 2 and 4), passage 2 diabetic cells from the same BBDP rat with extended T1DM that most readily expanded to passage 2 were used.

### Cell proliferation and β-galactosidase staining

BMSCs and PDCs were plated in six-well tissue culture plates (*n* = 3) with an initial seeding density of 20,000 cells/well, and cultured in high glucose (25 mM) or normal glucose (5.5 mM) expansion media for cell counting up to 7 days, using a separate plate for each time point. In a parallel set of experiments (*n* = 2), cells were fixed and stained on days 1, 4, and 6 for β-galactosidase activity as an indicator of senescence per the literature protocol [34].

### Osteogenic and adipogenic differentiation and growth factor rescue

BMSCs and PDCs were first cultured in high or normal glucose expansion media (*n* = 3) until 70–90% confluency before being switched to either osteogenic media (high or normal glucose expansion media supplemented with 10 nM dexamethasone, 20 mM β-glycerol phosphate, and 50 μM 1-ascorbic acid 2-phosphate) or adipogenic media (expansion media supplemented with 0.5 μM dexamethasone, 0.5 μM isobutylmethylxanthine, and 50 μM indomethacin). It should be noted that diabetic BMSCs were preconditioned in high glucose expansion media for a longer period than diabetic PDCs due to the extremely slow proliferation of the former (requiring a longer time for diabetic BMSCs to reach the desired confluency for differentiation induction). After 14 days of culture in the respective differentiation media, cells were fixed for alizarin red or oil red staining. In a parallel set of experiments, rhBMP-2/7 (45 or 300 ng), rhIGF-1 (15 or 100 ng), or a combination of both growth factors (45 ng rhBMP-2/7 + 15 ng rhIGF-1, or 300 ng rhBMP-2/7 + 100 ng rhIGF-1) were supplemented to the osteogenic media to assess their abilities to rescue impaired osteogenic differentiation of the diabetic cells in normal and high glucose cultures (*n* = 4). Staining was also carried out on cells cultured in both high and normal glucose expansion media (negative control). After photo-documentation of the alizarin red and oil red staining, the dye was released for quantitation per the literature protocol [32, 35].

### qPCR

Following 14 days of culture in high glucose osteogenic media with or without 300 ng rhBMP-2/7, total RNA was isolated from diabetic BMSCs and PDCs using TRIzol (Invitrogen, Carlsbad, CA, USA) and purified by Direct-Zol miniprep (Zymo Research, Irvine, CA, USA) following the vendor’s instructions. RNA was reverse transcribed into cDNA with SuperScript III Reverse Transcriptase (Invitrogen) according to the vendor’s instructions on a GeneAmp 2700 PCR system (Applied Biosystems, Foster City, CA, USA). qPCR (*n* = 3) was carried out on an Applied Biosystems 7500 Fast Real-Time PCR system with TaqMan Gene Expression Master Mix (Applied Biosystems) and inventoried TaqMan probes for bone gamma-carboxyglutamate protein (BGLAP), also known as osteocalcin (OCN), type 1 collagen (COL1), glucose transporter 1 (GLUT1), peroxisome proliferator-activated receptor gamma (PPARγ), and housekeeping gene glyceraldehyde 3-phosphate dehydrogenase (GAPDH). Gene expression was quantified using the delta–delta Ct method. Relative expression of each gene in a given cell type in the high glucose culture with rhBMP2/7 treatment is compared with the respective cultures without BMP-2/7 rescue (set as 1).

### Statistical analysis

**Fig. 1:**
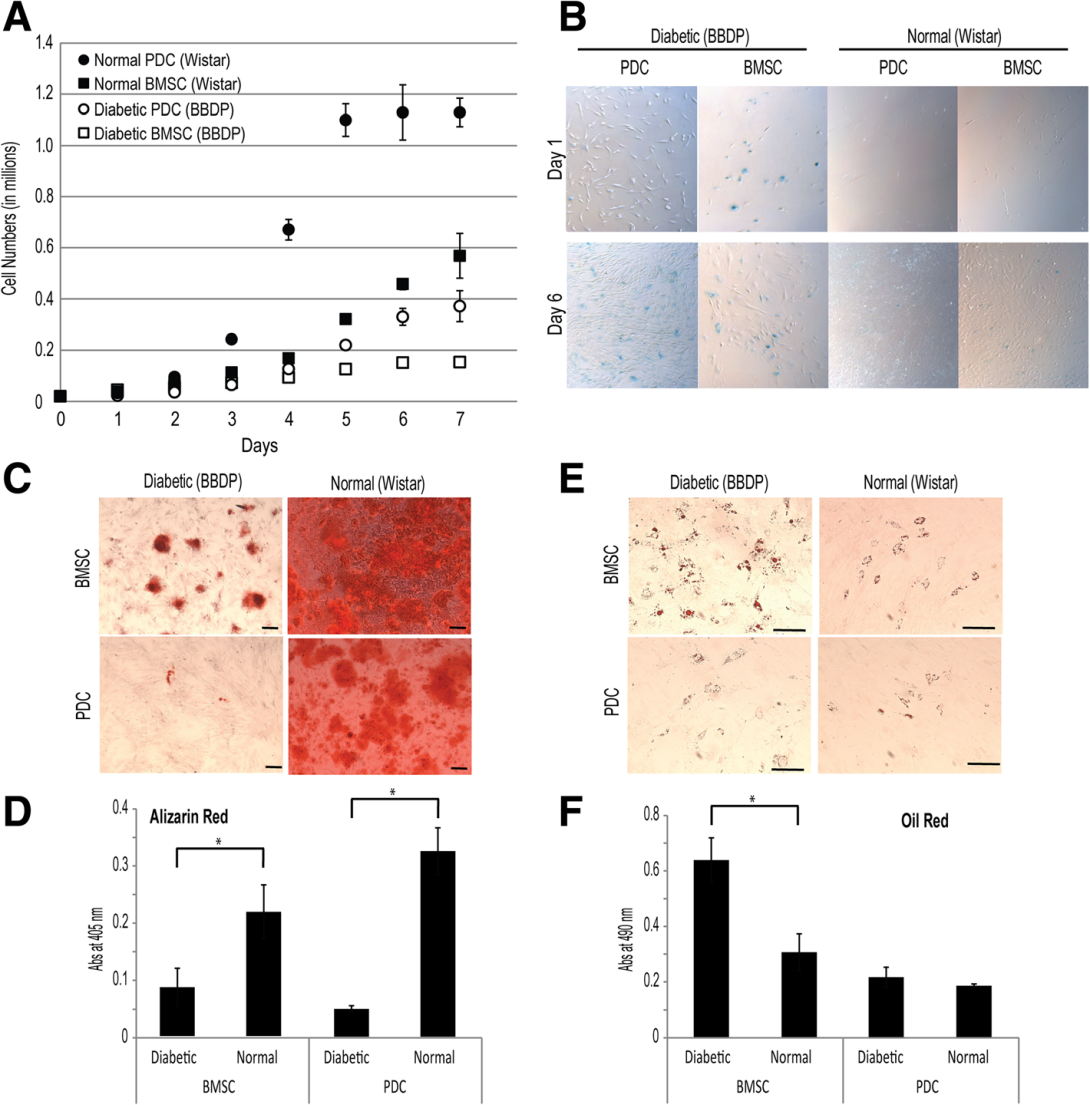
Impaired proliferation and osteogenic differentiation of diabetic BBDP BMSCs and PDCs in normal glucose (5.5 mM) cultures. **a** Cell proliferation (*n* = 3). **b** β-Galactosidase staining (*blue*) for senescent cells. Magnification: 50×; **c**, **d** Alizarin red staining and quantification (*n* = 3) after 14-day normal glucose osteogenic culture. *Scale bars* = 100 μm; **e**, **f** Oil red staining and quantification (*n* = 3) after 14-day adipogenic culture. *Scale bars* = 100 μm. *p < 0.05 (Student’s *t* test) for pairwise comparisons between cultures for a given cell type; *error bars*, standard deviation of the mean. All differences in cell numbers (**a**) between cultures at a given time point were significant (*p* < 0.05). *BBDP* Biobreeding Diabetes Prone/Worcester, *BMSC* bone marrow-derived stromal cell, *PDC* periosteum-derived cell

**Fig. 2:**
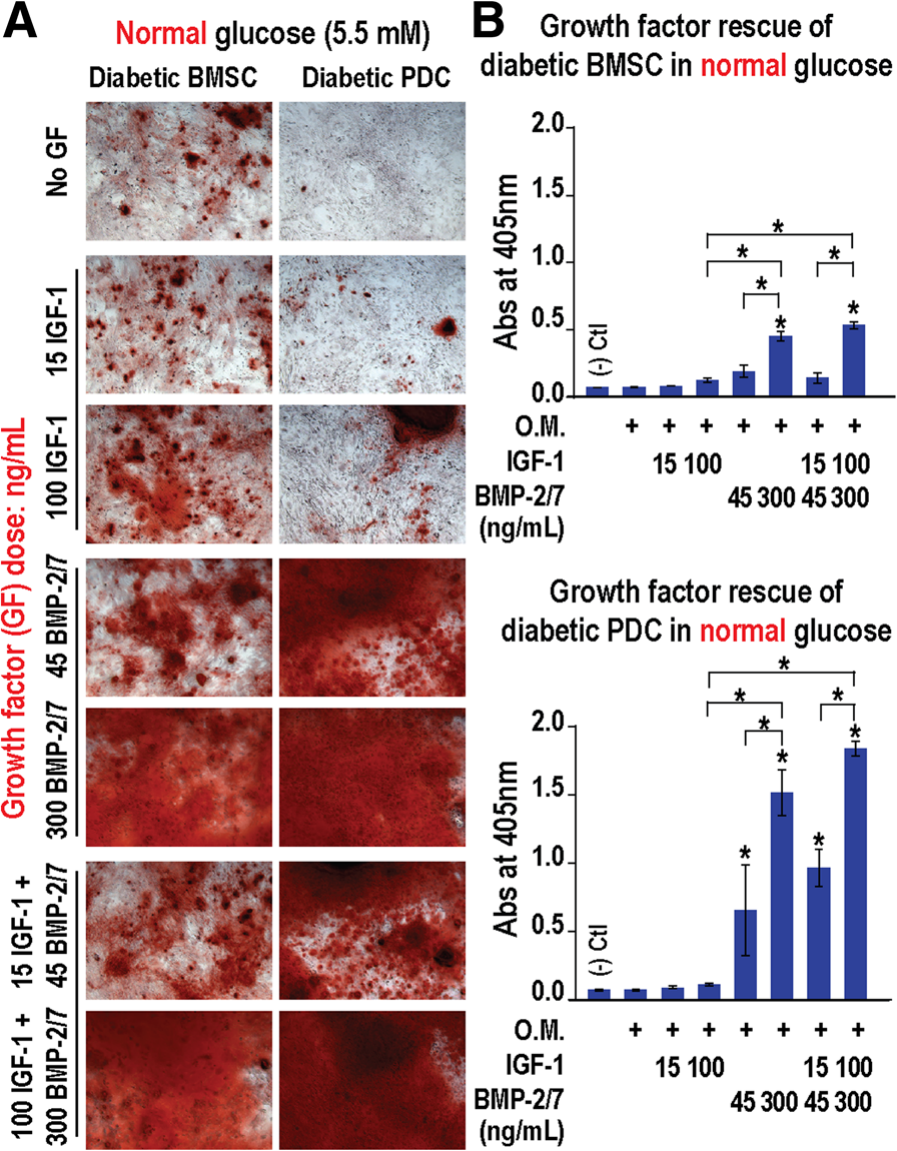
In-vitro rescue of impaired osteogenic differentiation of diabetic BMSCs and PDCs in normal glucose culture. **a** Optical micrographs (100×) and **b** quantification (*n* = 4) of alizarin red staining for osteogenesis of diabetic BMSCs and PDCs rescued by rhBMP-2/7 (45 or 300 ng/ml), rhIGF-1 (15 or 100 ng/ml), or their combinations after 2 weeks in normal glucose (5.5 mM) osteogenic media. *p < 0.05 (ANOVA) for pairwise comparisons with those cultured in osteogenic media without growth factor treatment (* *directly above the data bars*) or between single and dual growth factor treatments (* *above the brackets indicating specific data pairs compared*); *error bars*, standard deviation of the mean. *O.M. osteogenic media, BMP*-*2/7* bone morphogenetic protein-2/7 heterodimer, *BMSC* bone marrow-derived stromal cell, *IGF-1* insulin-like growth factor-1, *PDC* periosteum-derived cell

**Fig. 3:**
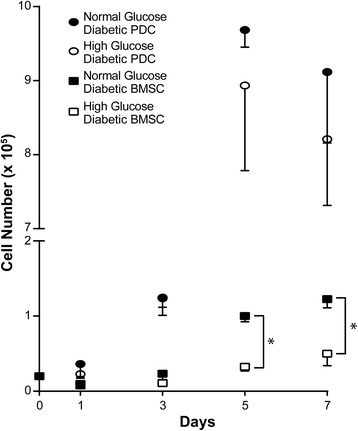
High glucose culture further impairs the proliferation of diabetic BMSCs and PDCs. Cells isolated from diabetic BBDP rats (*n* = 3) were cultured in normal (5.5 mM) or high (25 mM) glucose expansion media and were counted every other day for 1 week. *p < 0.05 (Student’s *t* test) for pairwise comparisons between high and normal glucose cultures for a given cell type at a given time point. *Error bars*, standard deviation of the mean. *BMSC* bone marrow-derived stromal cell, *PDC* periosteum-derived cell

**Fig. 4:**
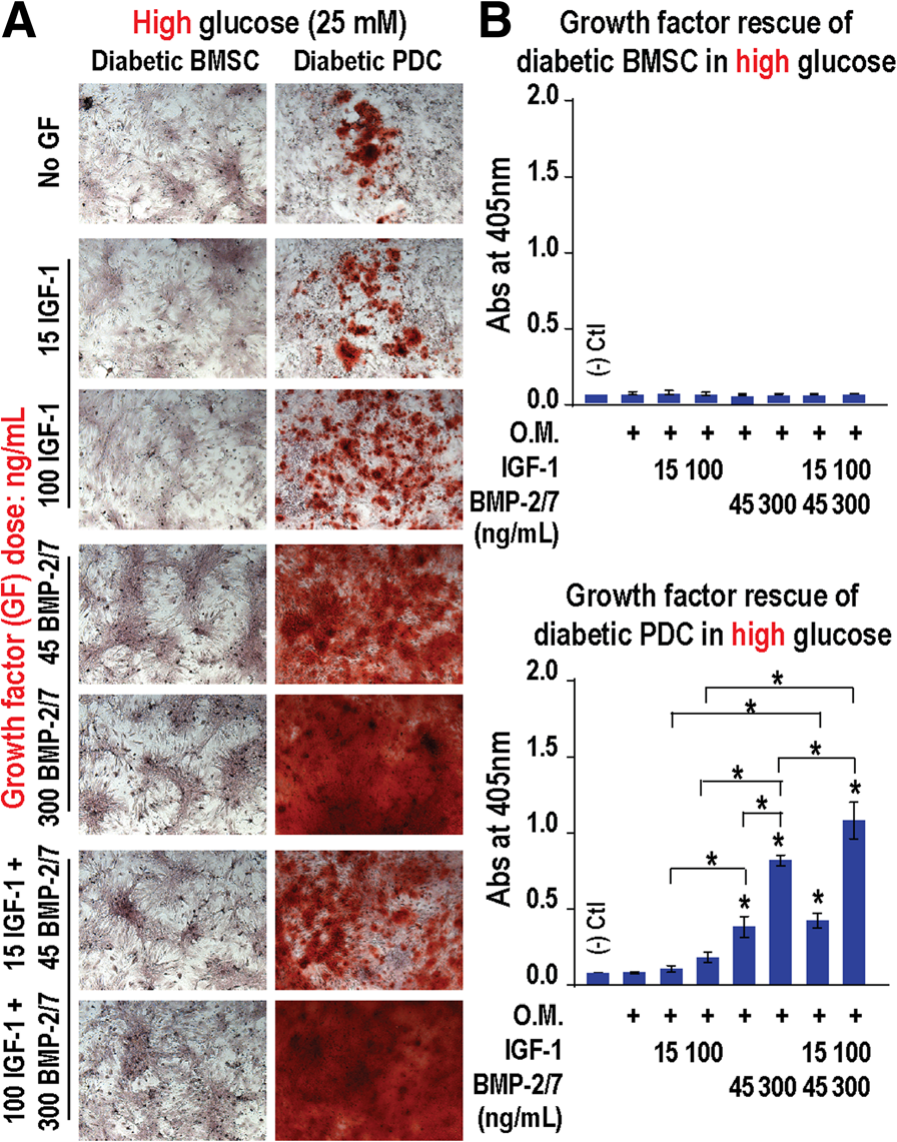
High glucose inhibits growth factor rescue of impaired osteogenic differentiation of diabetic BMSCs but not the rescue of osteogenesis of diabetic PDCs. **a** Optical micrographs (100 × x) and **b** quantification (*n* = 4) of alizarin red stain for osteogenesis of diabetic BMSCs and PDCs after 2-week culture in high glucose (25 mM) osteogenic media supplemented with rhBMP-2/7 (45 or 300 ng/ml), rhIGF-1 (15 or 100 ng/ml), or their combination. *p < 0.05 (ANOVA) for pairwise comparisons with that cultured in osteogenic media without growth factor treatment (* *directly above the data bars*) or between single and dual growth factor treatments (* *above the brackets indicating specific data pairs compared*); *error bars*, standard deviation of the mean. *O.M. osteogenic media, BMP*-*2/7* bone morphogenetic protein-2/7 heterodimer, *BMSC* bone marrow-derived stromal cell, *IGF-1* insulin-like growth factor-1, *PDC* periosteum-derived cell

**Fig. 5:**
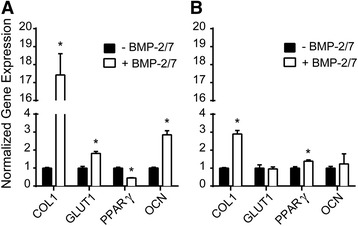
rhBMP-2/7 treatment (300 ng/ml) upregulates osteogenic gene expression and suppresses adipogenic gene expression in diabetic PDCs, but not in diabetic BMSCs, in high glucose (25 mM) osteogenic cultures. Relative Col1, OCN, GLUT1, and PPARγ expressions (*n* = 3) in **a** diabetic PDCs and **b** diabetic BMSCs in high glucose osteogenic cultures with rhBMP2/7 treatment are reported relative to that of the respective cultures without BMP-2/7 treatment (set to 1). *p < 0.05 (Student’s *t* test) for pairwise comparisons between cultures with and without growth factor rescue for a given cell type; *error bars*, standard deviation of the mean. *BMP*-*2/7* bone morphogenetic protein-2/7 heterodimer, *BMSC* bone marrow-derived stromal cell, *PDC* periosteum-derived cell, *Col 1* type 1 collagen, *GLUT1* glucose transporter 1, *PPARγ* peroxisome proliferator-activated receptor gamma, *OCN* osteocalcin

Student’s *t* test was applied for pairwise comparisons between diabetic and normal cells for a given culture induction (Fig. 1), between high and normal glucose cultures for a given diabetic progenitor at a given time point (Fig. 3), and between high glucose cultures with and without 300 ng/ml rhBMP-2/7 for a given diabetic cell type (Fig. 5). One-way analysis of variance (ANOVA) with Tukey post-hoc testing was applied for pairwise comparisons in single/combination growth factor rescue experiments (Figs 2 and 4). *p* < 0.05 was considered significant and all error bars represent the standard deviation of the mean.

## Results

### Proliferation and osteogenic differentiation of diabetic BMSCs and PDCs is impaired in normal glucose cultures

BMSCs and PDCs were isolated from the long bones of skeletally mature BBDP rats with established T1DM or normal (nondiabetic) Wistar rats for in-vitro characterizations. Both diabetic BMSCs and PDCs proliferated much slower than their nondiabetic controls in normal glucose (5.5 mM) culture (Fig. 1a) whereas PDCs proliferated more rapidly than their BMSC counterparts in general. Beta-gal staining revealed a larger senescent cell population (blue stain) in diabetic BMSC and PDC cultures as compared with their nondiabetic counterparts (Fig. 1b). Diabetic BMSCs and PDCs also exhibited impaired osteogenesis compared with their nondiabetic counterparts, as shown by significantly reduced alizarin red staining for mineral deposition after 2-week osteogenic induction (Fig. 1c, d). Although the osteogenesis of diabetic PDCs was severely suppressed, no significant difference in induced adipogenesis between diabetic and nondiabetic PDCs was observed by oil red staining (Fig. 1e, f). By contrast, diabetic BMSCs underwent far more potent adipogenesis than normal BMSCs upon culture induction (Fig. 1e, f).

### Impaired osteogenic differentiation of diabetic PDCs and BMSCs can be rescued by rhBMP-2/7 and rhBMP-2/7 + rhIGF-1 treatments in normal glucose culture

As IGF-1, an anabolic factor for skeletal growth [36], is reduced in T1DM patients with poor glycemic control [29–31], we examined whether supplementing osteogenic cultures of diabetic BMSCs and PDCs with potent osteogenic factor rhBMP-2/7 heterodimer [28] and/or rhIGF-1 may rescue their osteogenesis. In normal glucose (5.5 mM) osteogenic culture, rhBMP2/7 supplement enhanced the osteogenesis of diabetic BMSCs and PDCs in a dose-dependent manner, as indicated by alizarin red staining (Fig. 2a, b), to a level comparable to (for diabetic BMSCs with 45 ng/ml rhBMP-2/7) or beyond those of normal progenitors in response to osteogenic inductions in the absence of BMP-2/7 (normal controls in Fig. 1c, d). Although 100 ng/ml rhIGF-1 treatment alone also appeared to have a positive effect on the osteogenesis of diabetic progenitors, it did not result in statistically significant increase in alizarin red staining by 2 weeks, failing to rescue the impaired osteogenesis to the level of their normal progenitors. The combination of 100 ng/ml rhIGF-1 and 300 ng/ml rhBMP-2/7 was the most potent, resulting in over 6-fold and 26-fold increases in alizarin red staining after 2 weeks of osteogenic induction of diabetic BMSCs and diabetic PDCs, respectively (Fig. 2b). Such enhanced levels of alizarin red staining were also 2.5 and 5.7 times those observed with normal BMSCs and PDCs upon 2-week osteogenic induction in the absence of rhBMP-2/7 (Fig. 1d), respectively. However, we did not observe synergistic benefit of the dual growth factor treatment beyond the sum of the enhancements from respective single growth factor treatments.

### High glucose further impairs proliferation of diabetic BMSCs and inhibits the growth factor rescue of osteogenesis of diabetic BMSCs

Literature in-vitro studies examining BMSC dysfunctions induced by hyperglycemia typically utilize glucose concentrations ranging from 12 to 30 mM [37–39]. Here we show that the proliferation of diabetic BMSCs was further inhibited in high (25 mM) glucose cultures compared with normal glucose cultures (Fig. 3). The adopted 25 mM glucose concentration, nearly a 4.5-fold increase over the normal glucose (5.5 mM) culture, approximates the upper end of fold increases in blood glucose permitted in the T1DM BBDP rat maintenance designed to emulate poorly controlled glycemia. The proliferation of diabetic PDCs also slowed in high glucose culture (Fig. 3), but the difference was not statistically significant.

Compared with normal glucose osteogenic culture, high glucose osteogenic cultures appeared to further inhibit the osteogenesis of diabetic BMSCs, and such suppression could not be rescued by the supplementation of rhBMP-2/7 (45 or 300 ng/ml), rhIGF-1 (15 or 100 ng/ml), or their combination (Fig. 4a left, b top). By contrast, diabetic PDCs were able to respond to rhBMP-2/7 (45 or 300 ng/ml) treatment in a dose-dependent manner, resulting in statistically significant increases in alizarin red staining (Fig. 4a right, b bottom), exceeding that of normal PDCs upon osteogenic induction in normal glucose culture without rhBMP-2/7 (normal PDCs in Fig. 1d). The combination treatment, both 15 ng IGF-1 + 45 ng BMP-2/7 and 100 ng IGF-1 + 300 ng BMP-2/7, resulted in statistically significant further enhancement in alizarin red staining of diabetic PDCs in the high glucose culture, with the latter achieving >12-fold increase over that of the no-growth factor control, or >2-fold increase over that observed with normal PDCs in normal glucose osteogenic media without growth factors (Fig. 1d).

To further elucidate how diabetic progenitors differentially respond to the more potent BMP-2/7 treatment in high glucose osteogenic culture, gene expressions by diabetic PDCs and BMSCs were analyzed by qPCR after 2-week high glucose osteogenic inductions with/without 300 ng/ml rhBMP-2/7. Specifically, the expression of early stage osteogenesis marker COL1, osteoblast-secreted late stage marker OCN critical for matrix mineralization [40], GLUT1 responsible for glucose uptake (its expression found to positively correlates with osteogenesis [41]), and adipogenesis marker PPARγ responsible for fatty acid storage and glucose metabolism was quantified. Diabetic PDCs showed significantly higher (by approximately 17-fold, Fig. 5a) expression of osteogenesis marker COL1, which precedes matrix mineralization, in response to rhBMP-2/7 treatment in high glucose osteogenic culture, while diabetic BMSCs only responded to the growth factor supplement by a 3-fold increase in COL1 gene expression (Fig. 5b). The less effective rescue of COL1 expression in diabetic BMSC was accompanied by the complete failure in rescuing the gene expression of later-stage osteogenesis marker OCN (Fig. 5b), consistent with the lack of mineralized matrix staining by alizarin red (Fig. 4a, left). By contrast, rhBMP-2/7 treatment resulted in a statistically significant increase (~1.8-fold) in OCN gene expression in diabetic PDCs in the high glucose culture (Fig. 5a), agreeing with the successful rescue of the secretion of mineralized matrix shown by alizarin red staining (Fig. 4a, right). Meanwhile, GLUT1 expression was also enhanced in diabetic PDCs (~1.8-fold, Fig. 5a) with rhBMP-2/7 treatment but not in diabetic BMSCs (Fig. 5b). Finally, whereas the successful BMP-2/7 rescue of the osteogenesis of diabetic PDCs resulted in an expected decrease (2.2-fold, Fig. 5a) in the gene expression of adipogenesis marker PPARγ, the failed rescue in diabetic BMSCs was accompanied by a statistically significant increase (~1.4-fold) in PPARγ expression (Fig. 5b).

## Discussion

Although T1DM is known for orthopedic comorbidities such as decreased bone mineral density/volume, increased fracture risk, and impaired bone healing, effective therapeutic strategies for rescuing delayed T1DM bone healing are lacking. Whether and how different skeletal progenitor cells involved in long bone healing are differentially impaired in T1DM, thereby impacting their relative responsiveness to standard growth factor therapies, have not been carefully examined. In this study, a noticeable fraction of BMSCs and PDCs isolated from BBDP rats with newly acquired T1DM phenotype were found senescent. The diabetic BMSCs and PDCs exhibited significantly impaired in-vitro proliferation and underwent much poorer osteogenic differentiation, as revealed by alizarin red staining of mineralized matrices, upon culture induction compared with their nondiabetic counterparts, even when cultured in normal glucose media. Diabetic PDCs were found to proliferate slightly better than diabetic BMSCs, but underwent even poorer osteogenic differentiation than diabetic BMSCs in normal glucose osteogenic media. Although it was previously shown that restoring normal insulin levels could improve bone quality in T1DM patients [42], early intervention is needed to prevent detrimental “metabolic memory” caused by hyperglycemia [43]. Our observation that skeletal progenitors isolated from T1DM rats with poorly controlled blood glucose exhibit significant cellular impairments in normal glucose cultures supports that restoring normal blood glucose alone may not be sufficient in rescuing skeletal comorbidities of T1DM. Additionally, we found that the impaired osteogenesis of diabetic BMSCs was accompanied with more potent adipogenic differentiation upon culture induction, consistent with literature reports on the increased bone marrow adiposity in T1DM rodents [44]. There was no difference, however, in the adipogenesis of diabetic PDCs compared with nondiabetic PDCs. This observation suggests that mechanisms underlying the observed impairment of osteogenic differentiation of diabetic BMSCs and PDCs may differ.

Most earlier in-vitro studies on the cellular effects of high glucose were carried out with nondiabetic cells giving sometimes discrepant results [37, 39, 45–47], with some showing that high glucose compromises bone marrow cell growth and osteogenic differentiation or quality of mineral deposition [45–47]. Here we found that the proliferative potential of diabetic skeletal progenitors further diminished (marginally in diabetic PDCs, significantly in diabetic BMSCs) in high glucose culture. Interestingly, whereas high glucose also further suppressed the osteogenic differentiation of diabetic BMSCs, it appeared to have some positive effect on the osteogenesis of diabetic PDCs (although still significantly impaired compared with the osteogenesis of nondiabetic PDCs in normal glucose culture).

We then evaluated whether and how diabetic BMSCs and PDCs may differentially respond to the osteogenesis rescue in normal glucose and high glucose cultures by osteogenic factor BMP-2/7 heterodimer and/or IGF-1. We showed that both diabetic BMSCs and PDCs responded robustly to the rhBMP-2/7 rescue in a dose-dependent manner in normal glucose osteogenic culture, restoring their osteogenesis to the level comparable with (45 ng/ml treatment for diabetic BMSCs) or far beyond those of normal progenitors. By contrast, these diabetic progenitors responded only marginally to rhIGF-1 in normal glucose osteogenic culture. IGF-1 is an anabolic factor for skeletal growth known to be downregulated in poorly controlled T1DM [29–31]. It was previously shown to promote matrix mineralization by osteoblasts under high glucose conditions [48] and aid healing in calvarial defects in streptozocin-induced diabetic rats [49]. Here, we did not observe sufficient rescue of the impaired osteogenesis of diabetic BMSCs and PDCs by rhIGF-1 treatment alone even at doses consistent with the aforementioned literature reports. Although we showed that dual treatment of rhIGF-1 and rhBMP-2/7 resulted in statistically significant further enhancement in osteogenesis compared with rhBMP-2/7 treatment alone, no apparent synergistic benefits beyond the sum of enhancements of the respective single factor treatments was observed. In all cases, the diabetic PDCs responded more readily to these growth factor treatments than diabetic BMSCs in normal glucose culture.

In high glucose culture, only the diabetic PDCs responded to the rescue by rhBMP-2/7 or rhBMP-2/7 + rhIGF-1 in a dose-dependent manner while the significantly impaired osteogenesis of diabetic BMSCs remained completely suppressed and was unresponsive to the growth factor treatments. qPCR confirmed that diabetic PDCs responded to the rhBMP-2/7 rescue in high glucose osteogenic media by upregulations of early stage (COL1) and late stage (OCN) osteogenic markers and the glucose transporter GLUT1 known to positively correlate to osteoblastic differentiation [41], as well as the downregulation of adipogenic marker PPARγ. By contrast, diabetic BMSCs only exhibited a small increase in COL1 expression but no significant changes in OCN or GLUT1 expression after 2 weeks of rhBMP-2/7 treatment in high glucose osteogenic media. The adipogenic marker PPARγ expression in diabetic BMSCs even saw an increase upon rhBMP-2/7 treatment. These data further support the likelihood of differing mechanisms by which the two skeletal progenitor cells are impaired in a hyperglycemia environment, and thus the need to tailor the therapeutic strategies for T1DM bone healing based on specific cellular niches.

The effectiveness of rhBMP-2/7 in rescuing osteogenesis of diabetic PDCs in both normal and high glucose cultures demonstrated in this study supports the concept of local delivery of the osteogenic factor to the periosteal surface (e.g., via a resorbable mesh) to expedite retarded diabetic fracture healing, a significant clinical challenge among T1DM patients. It is worth investigating whether expedited diabetic fracture healing may be accomplished using clinically safe doses of the osteogenic factor with and without tight blood glucose control. Meanwhile, this study also underscores the unique challenge in rescuing the highly suppressed osteogenesis of diabetic BMSCs in the presence of persisted hyperglycemia. It is likely that impaired secretion of signaling molecules other than BMP-2, BMP-7, and IGF-1 in the bone marrow niche in T1DM will need to be rescued. For instance, increased secretion of inflammatory cytokines within T1DM bone marrow microenvironment [14, 29] and impaired angiogenesis [50, 51], a process known to be tightly coupled with osteogenesis during bone regeneration [52], has been reported in T1DM. It would be of interest to examine whether exogenous stromal derived factor SDF-1, shown to promote anti-inflammatory monocyte recruitment and stimulate vascular remodeling [53], and/or angiogenic factors (e.g., VEGF [54], sphingocine-1-phosphate [55]), could help more effectively rescue the osteogenesis of diabetic BMSCs in vitro and in vivo in the presence of hyperglycemia. Such an investigation will be particularly relevant for developing effective therapeutic strategies toward the regenerative repair of traumatic diabetic bony injuries (e.g., segmental bone loss) that disrupt the bone marrow niche, a condition less prevalent than fractures but far more clinically challenging.

## Conclusions

This study demonstrates that BMSCs and PDCs isolated from T1DM BBDP rats exhibited impaired proliferation and osteogenesis in vitro, and that these impairments were exacerbated in high glucose cultures. We showed that impaired osteogenesis of diabetic PDCs could be fully rescued by rhBMP-2/7 (45 or 300 ng/ml) with/without rhIGF-1 (15 or 100 ng/ml) in both normal and high glucose cultures in a dose-dependent manner. Diabetic BMSCs, however, only responded to the 300 ng/ml rhBMP-2/7 rescue with/without 100 ng rhIGF-1 in normal but not high glucose osteogenic cultures. IGF-1 alone was insufficient in rescuing the osteogenesis of either diabetic progenitor in the doses examined.

Our findings support the benefit of restoring normal blood glucose while delivering exogenous protein therapeutics compared with either approach alone in rescuing impaired T1DM bone healing. Furthermore, the findings that diabetic PDCs are more responsive than diabetic BMSCs to BMP-2/7 rescue in a hyperglycemia environment points to an exciting opportunity to rescue diabetic fracture healing, a prevalent clinical problem, via local delivery of the therapeutics to periosteal surfaces of the fracture. Finally, our study also underscores the unique challenge in rescuing the highly suppressed osteogenesis of diabetic BMSCs in the presence of hyperglycemia. Alternative/additional biochemical therapeutics tailored to the diabetic bone marrow niche will need to be identified to more effectively facilitate the regenerative repair of traumatic diabetic bony injuries that disrupt the bone marrow niche.

## Abbreviations

ANOVA: Analysis of variance
BBDP: Biobreeding Diabetes Prone/Worcester
BBDR: Biobreeding Diabetes Resistant/Worcester
BMSC: Bone marrow-derived stromal cell
Col 1: Type 1 collagen
DM: diabetes mellitus
FBS: Fetal bovine serum
GAPDH: Glyceraldehyde 3-phosphate dehydrogenase
GLUT1: Glucose transporter 1
OCN: Osteocalcin
PDC: Periosteum-derived cell
PPARγ: Peroxisome proliferator-activated receptor gamma
qPCR: Real-time polymerase chain reaction
rhBMP-2/7: Recombinant human bone morphogenetic protein-2/7 heterodimer
rhIGF-1: Recombinant human insulin-like growth factor-1
T1DM: Type 1 diabetes
α-MEM: Minimum Essential Medium alpha
